# Gender differences in ethmoid sinus morphology_ 3D reconstruction of computed tomographic images

**DOI:** 10.1186/s12880-024-01319-z

**Published:** 2024-06-10

**Authors:** Chi-Pin Hsu, Chih-Feng Lin, Chih-Chi Yang, Jeng-Ywan Jeng, Chang-Hung Huang

**Affiliations:** 1 Taiwan High Speed 3D Printing Research Center, National Taiwan University of Science and Technology, Taipei, Taiwan; 2 Department of Mechanical Engineering, Ming Chi University of Technology, New Taipei City, Taiwan; 3 Department of Otolaryngology Head and Neck Surgery, National Taiwan University Hospital, Taipei, Taiwan; 4 Department of Otolaryngology, Taichung Tzu Chi Hospital, Buddhist Tzu Chi Medical Foundation, Taichung, Taiwan; 5 Department of Mechanical Engineering, National Taiwan University of Science and Technology, Taipei, Taiwan; 6 Biomechanics Research Laboratory, Department of Medical Research, MacKay Memorial Hospital, New Taipei City, Taiwan; 7 School of Dentistry, National Yang Ming Chiao Tung University, Taipei, Taiwan

**Keywords:** Ethmoid sinus, Morphometrical analysis, 3D reconstruction, Computed tomography, Gender difference

## Abstract

**Background:**

The ethmoid sinus (ES) is a three-dimensional (3D) complex structure, a clear understanding of the ES anatomy is helpful to plan intranasal surgery. However, most prior studies use 2D measurements, which may not accurately depict the 3D structure. The current study measured the gender differences in ES morphology based on 3D reconstruction of computed tomography (CT) images.

**Methods:**

The 3D models were reconstructed using CT images. Twenty-one males and 15 females were enrolled in the study. The ES dimensions, including width, height and aspect ratio (AR) of each cutting-plane section, were measured at 10% increments along with the anteroposterior axis of the ES. The gender differences in the above parameters were further evaluated by an independent *t*-test.

**Results:**

The width of the ES for males is 12.0 ± 2.1 mm, which was significantly greater than that in females (10.0 ± 2.1 mm). The average height for males is 18.4 ± 3.5 mm, and 18.2 ± 3.4 mm for females. The AR of female (male) is around 0.56 (0.63) for the anterior ES and 0.66 (0.75) for the posterior. There are significant differences between genders in the parameters of width and AR (*p* < 0.05).

**Conclusion:**

This study found that the aspect ratio greatly varies along the length of ES, indicating that the cross-section of the ES in the anterior is closer to an elliptical shape and turns closer to a circular shape near its posterior. There is a significant difference between genders in width and aspect ratio. The results would be helpful to know the complex anatomic details of the ethmoid sinus.

## Introduction

The paranasal sinuses are an anatomically complex, interconnected system with air pockets that are connected to the nasal passages by cavities. The morphology of the nasal sinus varies significantly and is influenced by genetic diseases, infectious diseases, or environmental conditions [1–5]. Modern medical imaging technology allows the detection of sinus lesions, which improves the accuracy of measurement and allows appropriate planning for intranasal surgery [6–10].

**Fig. 1:**
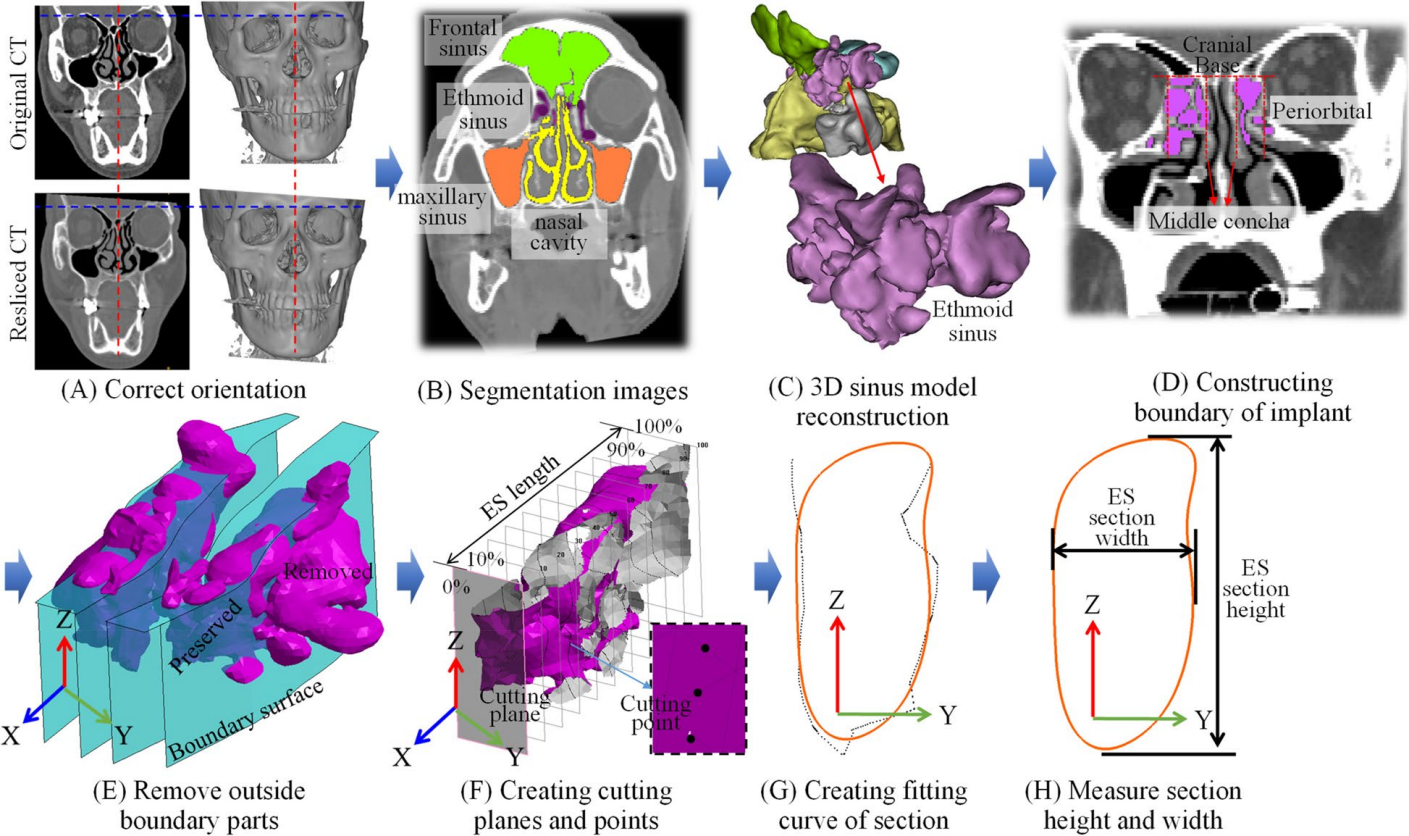
Methodology of the 3D model reconstruction and measurement process

The ethmoid sinus (ES) is a part of the paranasal sinuses and is located between the nose and eyes, and its boundaries include lamina papyracea of the orbit (lateral), middle and superior turbinates (medial) fovea ethmoidalis (superior), and superior border of inferior meatus (inferior) [11]. Furthermore, the ES is a common site for functional endoscopic sinus surgery [12–14]. The size and appearance of ES are important indicators of preoperative planning before surgery. Many morphometrical studies used 2D plane image technology to depict the 3D ES anatomy [15–21]. Those results varies study to study. The average length of the ES ranged from 21.3 ± 5.0 to 41.5 ± 3.8 mm, and the width ranged from 10.3 ± 3.0 to 17.3 ± 3.5 mm [16, 20, 21]. The possible reason results in the bias can be owing to the methods of CT scan and images post-processing, such as the positioning or orientation of the patients’ head when taking CT images [22, 23]. Measurement errors happen especially in the situations like head tilting during scanning or slice is non-orthogonal with sinus (Fig. 1A).

Moreover, traditional 2D image measures of the ES through X-rays and CT cannot have stereo observation on the complex anatomic structure of ES tunnels, but 3D model benefits in obtaining better visualization than 2D measures [24–26]. Besides, 3D model can be easily rotated and reoriented by changing the rotational axis or redefined the coordinate system through 3D reconstruction modeling technique, which have been used in the complex human skeletal structures [27–31].

Some morphological studies have reported racial and gender differences in sinus [15, 16, 20, 32]. One recent study showed that Chinese ES was about 16% wider than Caucasians. This study also reported that the ES width of Chinese was 13% higher in males than in females [20], but the opposite is true for Africans [15]. These studies showed that the anatomy of the sinus was affected by factors such as race, gender or the measurement method. Most of the data for previous studies was collected using 2D CT images. Although other studies used 3D measure strategies but they only calculated the volume [26, 32] or just used to classify the shape classifications [25, 33]. Three-dimensional data for the ES structure has yet to be collected.

Thus, current study attempted to measure the 3D structure of the ES by using a CT image reconstruction model. This model reconstruction and measurement method has been approved in the research team’s prior study when measuring the lower extremity of the human body [34]. The outline at each cutting-plane section of the ES was measured to determine the entire structure of the ES. The ES geometry for male and female subjects was also compared to determine the differences between genders in Asian populations. It is hypothesized that the ES of male subjects is significantly larger than that of females.

## Materials and methods

This study included subjects from patients with nasolacrimal duct stenosis for dacryocystorhinostomy, pituitary or skull base tumors who underwent surgery, and those who complained of olfactory dysfunction but demonstrated normal sinonasal findings on their computed tomography scans. Patients with previous sinonasal surgery for anatomical or inflammatory conditions, head and neck cancer received radiotherapy, trauma, orthognathic surgery, or congenital sinonasal disorders were excluded from the enrollment. A total of 36 subjects (21 males and 15 females) were included in the study which was approved by the Institutional Review Board (201912187RINA). The average age of the subjects is 55.4 years (21 ~ 99): a mean of 52.0 years old for males and 59.2 for females. All subjects had intact sinuses on both sides so a total of 72 sinus data points (42 male and 30 female data) were analyzed.

### Study design

The measurement process and study design for this study is shown in Fig. 1. CT scans were performed using a multi-slice CT machine (Sensation 16 SLICE, SIEMENS, Memphis, USA) with a 1 mm slice thickness (120 kV, 20 mA). Image segmentation and reconstruction used OOOPDS (Main Orthopedic Biotechnology Co., Ltd., Taichung City, Taiwan). The image was first correctly oriented using the criteria for the Frankfurt plane [35]. The ethmoid sinus (ES) level and the coordinates were then defined (Fig. 1A). The sinus cavity region is used to define the appearance of the sinus. The sphenoid sinus (SS), frontal sinus (FS), maxillary sinus (MS) and nasal cavity were segmented together with the ethmoid sinus, which are located at the specified level and coordinates and are in close proximity (Fig. 1B). The sinus cavity is reconstructed as a 3D model, and a boundary surface is defined by the standard anatomy of the ES (Fig. 1C-E). This study focuses on the standard anatomical narrative of the ES area [11, 36]. The ES model then removed the area outside the boundary surface and constructs cutting plane increments of 10% in the anterior–posterior (AP) direction for the remaining ES models (Fig. 1F). The cutting plane and the overlapping position of the ES model is the section contour of the ES. The total length of the ES and the width and height of each section were measured and the differences between genders were further determined (Fig. 1F).

### Definition of the coordinate system and the correct orientation of the images

**Fig. 2:**
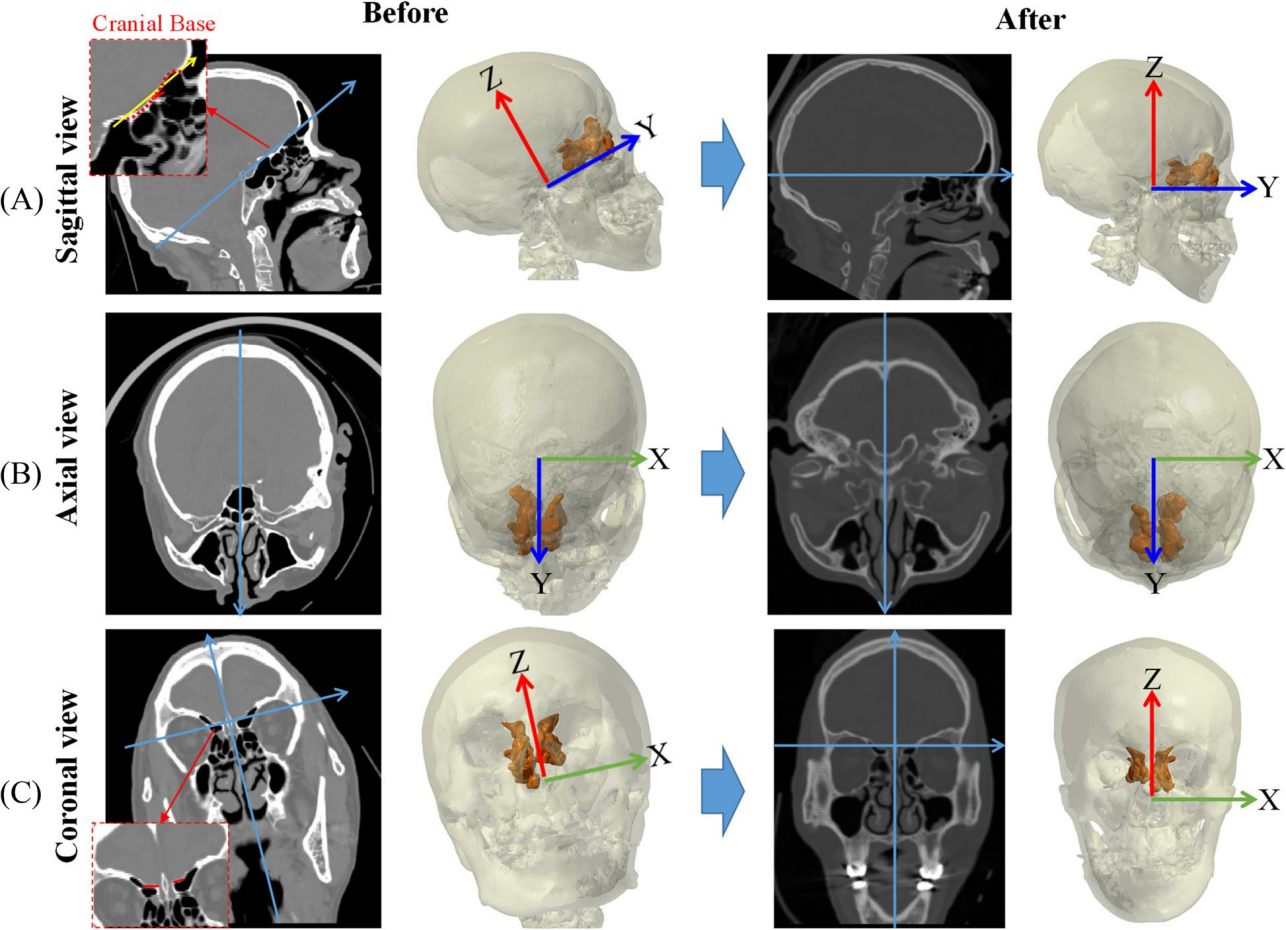
Coordinate definition and correction of image orientation: **A** sagittal view, **B** axial view and **C** coronal view

Different image orientations produce errors in measurement so the coordinate system is defined initially. The coordinate definition uses the Frankfurt plane [35]. The cranial base in the sagittal view is used as the horizontal basis to correct the Y-axis rotation of the model (Fig. 2A). The Z-axis rotation is corrected using the nasal septum in the axial view as the vertical basis (Fig. 2B). The X-axis rotation of the model is then corrected using the cranial base and the nasal septum in the coronal view as the vertical and horizontal basis (Fig. 2C).

### Defining and constructing ES model and boundaries

The sphenoid, frontal, maxillary, ES and nasal cavities are segmented and reconstructed for this study (Fig. 1B and C). The correctness of the image segmentation was confirmed by two ENT surgeons who are co-authors of this study (Lin CF and Yang CC). The boundaries of the ES were defined based on standard anatomical descriptions (Fig. 1D) [11, 36]. The upper boundary is defined as the cranial base, the outer boundary is periorbital and the inner boundary is middle turbinate. The boundary of each section is reconstructed as a boundary surface of the 3D model (Fig. 1E). The irrelevant area beyond the boundary surface is removed, so the remaining area of ES is the target for subsequent measurements (Fig. 1F). All cavities of the sinuses are reconstructed as 3D models and the correctness of the division was verified by two coauthors to minimize the error in boundary definition.

### Measurement of the ethmoid sinus

Reverse engineering software RevCAD V1.50 (PouYuen Tech Corp., Taoyuan, Taiwan) was used to create cutting planes (Fig. 1F). In order to analyze the complex anatomical appearance of ES objectively, the total length of the ES is used as a benchmark and the starting plane is through the frontmost point of the ES and perpendicular to the Y axis. Cutting planes are established at every 10% increment of the total length. The vertical distance between the 0% and 100% cutting planes is defined as the ES length (L) (Fig. 1F).

The intersection of the cutting planes and ES are defined as cutting points. The closed curve is calculated using a B-spline curve fitting algorithm with the cutting points (Fig. 1G) [37]. The distance from maximum Z_*i*_ to minimum Z_*i*_ for the closed curve is the height (H_*i*_) of this ES section (Fig. 1H), where *i* = 0%, 10%, 20%,…, and 100%. The distance from maximum Y_*i*_ to minimum Y_*i*_ for the closed curve is the width (W_*i*_) of the ES section (Fig. 1H). For this study, the aspect ratio (AR) of width to height for each ES section is defined as AR_*i*_ = W_*i*_*/*H_*i*_, AR_*i*_ and is used to determine the change in shape for each section of the chamber, which clearly defines the anatomy.

### Statistical analysis

To ensure that the data is normally distributed, the Kolmogorov–Smirnov test was used and proven for *p* > 0.05 in parametric statistics. G-power 3.1 was used for a post hoc power analysis of gender differences, with α = 0.05, and the calculated statistical power is 97%. Statistical data is analyzed using SPSS (version 20, SPSS Inc., Chicago, IL, USA) and independent *t*-tests are used to determine the significance of gender differences. A value of *p* < 0.05 indicates statistical significance.

## Results

**Fig. 3:**
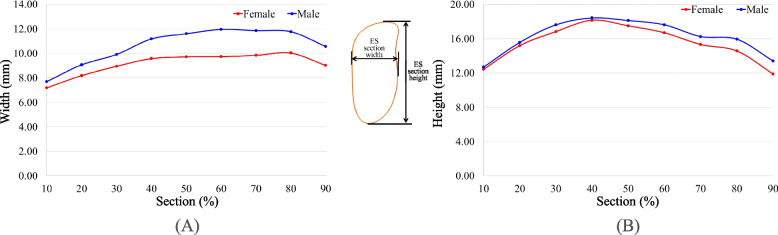
Average width and height of the ES for males and females in each section: **A** width and **B** height

The average ES length for the male group is 40.1 ± 5.0 mm and for the female group is 41.3 ± 5.0 mm. There is no significant difference between genders (*p* = 0.140) (Table 1). As for the average width and height of the ES for males and females in each section (Fig. 3). The maximum width of the male ES is W_60_ = 12.0 ± 2.1 mm and the maximum width of the female ES is W_80_ = 10.0 ± 2.1 mm. The male ES is significantly wider than the female ES. Except for W_10_ (*p* = 0.064), there is a statistically significant difference in most of the sections (*p* < 0.05). The male and female ES has a maximum height at H_40_ (18.4 ± 3.5 mm and 18.2 ± 3.4 mm). The maximum height for the male group is slightly greater than that for the female group for all ES heights, but the only significant difference is at H_60_ (*p* = 0.045) and H_80_ (*p* = 0.048).

**Table 1:** Size comparison of ethmoid sinus in previous 2D measurements studies and this 3D measurements study

Measurement method		Length (mm)			Width (mm)			Height (mm)		
		M	F	*p*	M	F	*p*	M	F	*p*
Ameye SA’s study [16]	2D CT	41.5 ± 3.8	41.2 ± 4.6	NA	Anterior: 11.6 ± 1.4 Posterior: 16.4 ± 3.9	Anterior: 11.2 ± 2.0 Posterior: 17.3 ± 3.5	NA	30.2 ± 6.0	29.7 ± 5.2	NA
Chan MA’s study [20]	2D CT	37.6 ± 3.4	37.0 ± 4.1	0.445	11.4 ± 1.9	10.1 ± 1.9	0.003	NA	NA	NA
Abdalla MA’s study [21]	2D CT	21.3 ± 5.0	23.5 ± 5.1	NA	10.3 ± 3.0	8.6 ± 3.0	NA	21.3 ± 3.6	20.3 ± 3.6	NA
Current study	3D and section analysis	40.1 ± 5.0	41.3 ± 5.0	0.140	Anterior: 9.9 ± 2.4 Posterior: 11.6 ± 2.4 W_80_:^a^ 11.8 ± 2.7	Anterior: 8.7 ± 1.9 Posterior: 9.7 ± 2.0 W_80_: 10.0 ± 2.1	0.000 0.000	H_40_:^b^ 18.4 ± 3.5 H_avg._: 16.2 ± 4.2	H_40_: 18.2 ± 3.4 H_avg._: 15.4 ± 3.9	0.364

^a^The ES width for Chan MA’s study is defined as the largest width of the ethmoid sinus in the axial CT image. This position is about W_80_ for this study

^b^The largest ES height is about H_40_ for this study

**Fig. 4:**
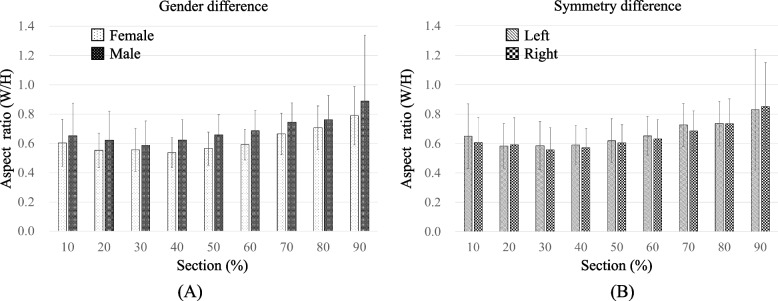
Average aspect ratio of the ES in different sections: **A** for gender difference and **B** for symmetry difference

The largest and smallest aspect ratio (AR) values for the male group are AR_*90*_ = 0.9 ± 0.5 and AR_*20*_ = 0.6 ± 0.2, respectively (Fig. 4A), and for the female group, these values are AR_90_ = 0.8 ± 0.2 and AR_30_ = 0.5 ± 0.1, respectively. The AR values for the male group are larger than those for the female group. There is a significant difference from AR_*40*_ to AR_*70*_ but the AR value for the anterior section (AR_*10*_- AR_*50*_) for the male group is 19.4% smaller on average than that for the posterior section (AR_*50*_- AR_*90*_). The anterior section for the female group is 18.3% smaller on average than the AR value for the posterior section. There are significant differences between AR_10_ vs. AR_90_, AR_20_ vs. AR_80_, AR_30_ vs. AR_70_, and AR_40_ vs. AR_60_. The AR for the left ES is slightly greater than the value for the right side (Fig. 4B) but there is no significant difference (*p* = 0.26).

**Fig. 5:**
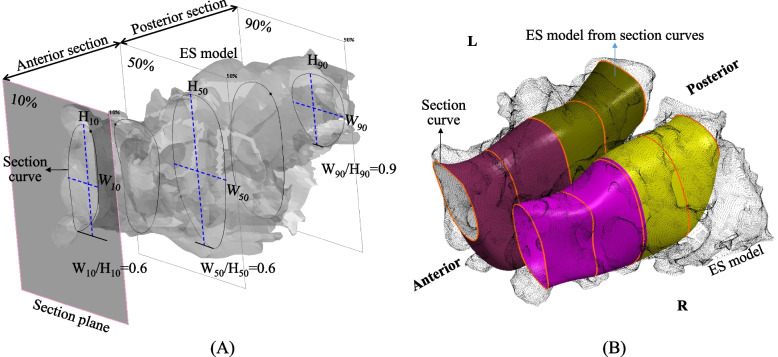
Diagram showing the shape of the ES in the anterior and posterior sections (**A**) the AR of the ES in the anterior and posterior sections (**B**) reconstructing ES model according section curve

The AR of the cross-section is about 0.6 for the anterior sections, so the cross-section of the ES in the anterior sections is close to an elliptical cylinder (Fig. 5A). However, the AR values for the cross-section in the posterior part are about 0.7–0.9, so the cross-sections in the posterior parts is almost circular.

## Discussion

**Fig. 6:**
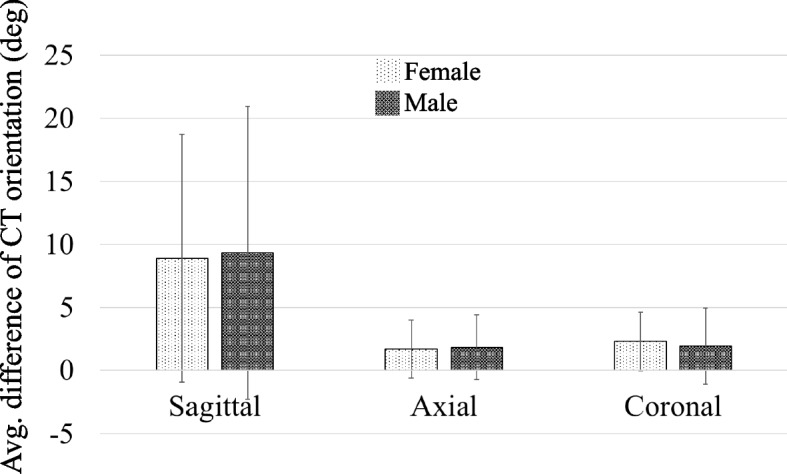
The average orientation difference between the original CT and the corrected model

The ethmoid sinus is the most anatomically complex part of the paranasal sinuses. It is created by the ethmoid bone, forming multiple pyramidal air cells. For this study, the 3D model is reconstructed to measure the detail of the ES section. This study allows a more accurate description of the anatomy of the ES than the 2D measurements. Most studies use 2D or planar images to measure the length of ES [15–21]. Those studies use one image that is the uppermost edge of the optical canal in several axial-view images and measure the length of ES using this image. However, the orientation of the 2D image is affected by the subject’s posture during the CT scan (Fig. 2). In standard scanning process, subjects used a head fixation device when taking CT images. However, the average orientation difference between the original CT and the corrected model is 9.1° ± 10.6, 1.8° ± 2.4 and 2.1° ± 2.7 in the sagittal, axial and coronal plane (Fig. 6). Especially in the sagittal plane, the orientation difference is 3.3 times and 4.2 times than the axial and coronal plane, respectively.

Two-dimensional (2D) imaging has notable limitations in medical contexts, particularly in assessing complex sinus anatomy. A primary issue is the flat representation, which lacks depth and fails to accurately depict spatial relationships between anatomical structures. Additionally, overlapping structures in 2D images make it challenging to distinguish boundaries and relationships, obscuring crucial details necessary for precise surgical planning. Measurement errors are another concern with 2D imaging. The accuracy of measurements can be compromised by the angle at which images are taken, resulting in inaccurate dimensions and shapes of anatomical structures. Without depth perception, it is difficult to understand the true extent and position of abnormalities such as polyps or tumors. Besides, 2D images are typically analyzed in a single plane (axial, coronal, or sagittal), restricting the ability to understand complex anatomy that exists across multiple planes simultaneously.

Regarding three-dimensional (3D) imaging, it offers significant advantages for medical applications, especially in understanding complex sinus anatomy. A true 3D representation provides an accurate depiction of spatial relationships and structure depth, crucial for navigating intricate anatomical regions during surgery. With clear visualization from any angle, the risk of missing critical details is reduced. Surgeons can also simulate surgeries, practice procedures, and devise effective approaches using 3D models, leading to better outcomes and shorter operative times. These models allow for personalized surgical plans tailored to each patient’s unique anatomy, improving precision and safety. 3D measurements are consistent and precise, unaffected by imaging angles, reducing the risk of errors related to anatomical misinterpretations. Detailed volume assessments of anatomical structures and pathological lesions are possible, aiding in disease extent evaluation and intervention planning.

While 2D measurements provide useful information, they fall short in accurately representing the complex and three-dimensional nature of sinus anatomy. 3D measurements and reconstructions offer a more comprehensive and precise understanding, significantly enhancing preoperative planning, surgical precision, and patient outcomes. The shift from 2 to 3D imaging represents a major advancement in medical imaging, particularly in fields requiring intricate anatomical navigation such as sinus surgery. Researches have shown that keeping consistently positioning and orientation of the patient’s head when taking CT images is a great challenge [22, 23]. It’s should be noted that the CT images are not definitely orthogonal to the standard landmarks (Frankfurt plane) of the ES structure. Accuracy ES dimension obtained from 2D image must be reoriented to ensure the measurement are done under the same coordinates. However, the reorientation for plane images is a complex process, and it is difficult to complete the reorientation process in traditional 2D image researches [15–21]. This study redefined the coordinate system based on the Frankfurt plane [35] and this process eliminated the possible deviations when measuring the dimensions of complex structures. Therefore, the current study can provide more relatively reasonable results and better visualization of the ethmoid sinus compared to previous studies [15–18].

The height of ES is an important measure. A complete sinus model was used in this study, so the anatomical location and identification of the ES region is clearer. The height of each section of the ES and the width of the ES were measured continuously at every 10% interval of the ES length (Figs. 1H and 4). The results showed that the average height of the ES for a Chinese male was 13.7 ± 6.6 mm and for a Chinese female is 13.0 ± 6.2 mm. No significant difference in the height of the ES was found for male and female groups (*p* > 0.05) but there is a statistically significant difference in width. The average width of the ES for a Chinese male was 9.1 ± 4.1 mm and for a Chinese female was 7.8 ± 3.4 mm. The male ES is about 20.0% larger than that of the female.

The difference between the measurement results for this study and those of other studies, in terms of length, width and height are about -47%‒3%, -14%‒78% and 12%‒64%, respectively (Table 1). The results show that there is some  discrepancy   between the results of this study and those of other literature, possibly due to differences in 3D and 2D measurement methods, measure locations, or ethnic differences.

The reconstruction and measurement methods in this study have been recognized in previous studies by measuring the morphology of the femoral condyle [34]. This is the first study to measure the dimensions and aspect ratio of a detailed ES structure using a 3D reconstruction model and a cutting plane method. The shape of an ES section is defined using the aspect ratio of the width to the height of the ES. If AR = 1, the shape is a circle and if AR < 1, it is closer to an ellipse. The results of this study showed that the average shape of ES is closer to an ellipse, especially for the anterior ES (AR_10_-AR_50_), for which the AR value is about 0.60, and the posterior ES (AR_50_-AR_90_), for which the AR value is about 0.74 (Fig. 5A). The aspect ratio for the end of the ES (AR_80_-AR_90_) is about 0.9, so posterior ES is approximately like a circle in the final section. As for the AR value in male ES is significantly greater than that for a female, particularly in the range of AR_40_- AR_70_.

A large number of Asians suffer from chronic sinusitis [38, 39] and may require stents to prevent restenosis after ES surgery [40–42]. Determining the 3D structure of an ES cavity allows more accurate planning for nasal surgery. The current ES stent has a circular section after it is deployed [40], but the anterior part should be elliptical and the posterior part should be almost circular. So, it does not fit the cavity shape. Our previous biomechanics study [43] analyzed the contact behavior of a nasal stent that was deployed to the ES cavity and showed that there was a significant rise of contact stress on the inside of the ES and a part of the stent material has caused permanent plastic deformation possibly due to mismatch between the stent and nasal cavity. If the shape of the implant deviates too much from the shape of the ES, excessive stress can occur and the ES can become loose [43]. The design of a commercial stent should ensure optimal stent-ES contact in the ES cavity. The section curve and the aspect ratio of the ES can provide helpful information for the improvement of the nasal device (Fig. 5B).

This study has some limitations should be mentioned here. First, the subject number is not big enough. The current study primarily developed a new measurement method to obtain 3D results in this stage and we will keep enrolling more data in the near future. Second, in order to obtain the most typical measurement data, this study only included normal anatomy cases. However, the morphology of ES may differ from the typical one after FESS. Third, the standard anatomy of the ES defined the upper (cranial base), outer (periorbital), and inner boundary (middle turbinate), but not inferior boundary [11, 36]. Therefore, this study defined the inferior boundary by the lowest point of section curve (Fig. 1G). Finally, there are multiple ways to demarcate and measure the morphology but it is difficult to define the boundaries of these complex air sacs. In addition to the limitations mentioned above, the current study has put new information to know the continuous change of the ES.

## Conclusion

This three-dimensional morphological analysis showed that the sinus cavity was constantly changing from anterior to posterior. The ethmoid sinuses are significantly wider in males than in females. Aspect ratio analysis showed that the ethmoid sinus near the anterior part was closer to the oval, but the shape of the posterior part resembled a circle. The aspect ratio of the middle part of the ethmoid sinus is significantly greater in males than in females.

## Data Availability

Data availability The datasets generated and/or analyzed during the current study are not publicly available due to the nature of this study, but are available from the corresponding author on reasonable request.
